# Developing a nomogram based on multiparametric magnetic resonance imaging for forecasting high-grade prostate cancer to reduce unnecessary biopsies within the prostate-specific antigen gray zone

**DOI:** 10.1186/s12880-017-0184-x

**Published:** 2017-02-01

**Authors:** Xiang-ke Niu, Jun Li, Susant Kumar Das, Yan Xiong, Chao-bing Yang, Tao Peng

**Affiliations:** 1 Department of Radiology, Affiliated Hospital of Chengdu University, Chengdu, 610081 China; 2 Department of General Surgery, Affiliated Hospital of Chengdu University, No. 82 2nd North Section of Second Ring Road, Chengdu, Sichuan 610081 China; 3 Department of Intervention Radiology, Tenth People’s Hospital of Tongji University, Shanghai, 200072 China

**Keywords:** Prostate cancer, Prostate-specific antigen, Magnetic Resonance Imaging, PI-RADS, Nomogram

## Abstract

**Background:**

Since 1980s the application of Prostate specific antigen (PSA) brought the revolution in prostate cancer diagnosis. However, it is important to underline that PSA is not the ideal screening tool due to its low specificity, which leads to the possible biopsy for the patient without High-grade prostate cancer (HGPCa). Therefore, the aim of this study was to establish a predictive nomogram for HGPCa in patients with PSA 4–10 ng/ml based on Prostate Imaging Reporting and Data System version 2 (PI-RADS v2), MRI-based prostate volume (PV), MRI-based PV-adjusted Prostate Specific Antigen Density (adjusted-PSAD) and other traditional classical parameters.

**Methods:**

Between January 2014 and September 2015, Of 151 men who were eligible for analysis were formed the training cohort. A prediction model for HGPCa was built by using backward logistic regression and was presented on a nomogram. The prediction model was evaluated by a validation cohort between October 2015 and October 2016 (*n* = 74). The relationship between the nomogram-based risk-score as well as other parameters with Gleason score (GS) was evaluated. All patients underwent 12-core systematic biopsy and at least one core targeted biopsy with transrectal ultrasonographic guidance.

**Results:**

The multivariate analysis revealed that patient age, PI-RADS v2 score and adjusted-PSAD were independent predictors for HGPCa. Logistic regression (LR) model had a larger AUC as compared with other parameters alone. The most discriminative cutoff value for LR model was 0.36, the sensitivity, specificity, positive predictive value and negative predictive value were 87.3, 78.4, 76.3, and 90.4%, respectively and the diagnostic performance measures retained similar values in the validation cohort (AUC 0.82 [95% CI, 0.76–0.89]). For all patients with HGPCa (*n* = 50), adjusted-PSAD and nomogram-based risk-score were positively correlated with the GS of HGPCa in PSA gray zone (*r* = 0.455, *P =* 0.002 and *r* = 0.509, *P =* 0.001, respectively).

**Conclusion:**

The nomogram based on multiparametric magnetic resonance imaging (mp-MRI) for forecasting HGPCa is effective, which could reduce unnecessary prostate biopsies in patients with PSA 4–10 ng/ml and nomogram-based risk-score could provide a more robust parameter of assessing the aggressiveness of HGPCa in PSA gray zone.

## Background

Prostate cancer (PCa) is the third leading cause of cancer death among men worldwide [1]. The introduction of prostate-specific antigen (PSA) in selecting men for prostate biopsy leads to earlier detection of prostate cancer (PCa) and, perhaps, a reduction in PCa-specific mortality [2]. However, there has been a steady rise in the detection of low-grade PCa (commonly referred to as over-diagnosis) and subsequent overtreatment [3]. This problem is attributable to the poor sensitivity and specificity profile of PSA. This is particularly the case in a PSA gray zone (4–10.0 ng/ml), at which 65–70% of men have a negative biopsy result [4]. Men with indolent disease who undergo treatment may experience complications without reducing their risk of dying from PCa [5].

Some PSA evolutional indexes are widely used clinically, such as free/total PSA ratio (PSA f/t ratio) and PSA density (PSAD). However, they are all provincial because of their dependence on PSA [6]. Furthermore, several other advanced attempts have been performed, such as 4 K score [7] and messenger RNA (mRNA) [8]. Though these models based on these new tests might be useful, the unavailable parameters limit the application. Nowadays, the growing availability of Multiparametric magnetic resonance imaging (mp-MRI) and increased standardisation has increased the role of prostate MRI in detecting of prostate cancer [9]. Prostate Imaging Reporting and Data System version 2 (PI-RADS v2), which was released online in the form of a 55-page document in December 2014, the overall five-point scale used in PI- RADS v2 is not designed for every cancer but for high-grade prostate cancer (HGPCa) that may require further work-up or target biopsy [10]. Therefore, the aim of this study was to develop a model combining prostate mp-MRI with traditional clinical risk factors that could be used to identify patients accurately with HGPCa (Gleason score ≥ 7) on reduction of unnecessary prostate biopsies in PSA gray zone.

## Methods

### Subjects

The retrospective study was approved by the regional ethical board of the Affiliated Hospital of Chengdu University. Informed written consent was obtained from all subjects prior to inclusion in the study. Inclusion criteria were suspicion of PCa owing to increased PSA levels combined with a suspicious abnormality at MR imaging eligible for target biopsy (TB) and available clinical data such as PSA level, DRE and TRUS results. Exclusion criteria were as follows: the patient had a history of prostate biopsy, the patient had benign prostatic hypertrophy treated with a 5a-reductase inhibitor, and the patient had a contraindication to transrectal US-guided biopsy (eg, anorectal stenosis). Two temporally separated patient cohorts were identified: January 2014 to September 2015 (training cohort) and October 2015 to October 2016 (validation cohort). In total, 225 consecutive patients with prebiopsy PSA between 4 ng/ml and 10 ng/ml were finally enrolled for evaluation.

### MRI protocol

Subjects underwent mp-MRI using a 3.0 T MR imager (Tim Trio, Siemens Healthcare, Erlangen, Germany) with a six-channel phased-array body coil. To suppress bowel peristalsis all patients received 20 mg butylscopolamine (Buscopan; Boehringer, Ingelheim, Germany) intravenously. The main imaging protocols included high-resolution axial T2WI, DWI, and DCE-MRI. An axial fat saturation T2W turbo spin echo (TSE) sequence (TR/TE, 4000/100 ms; slice thickness, 3 mm; no interslice gap; echo train length, 23; averages, two; field of view [FOV], 200 × 200 mm) were acquired. Diffusion-weighted imaging (DWI) was acquired using a single-shot echoplanar imaging (EPI) sequence. The slice thickness was 3.0 mm with no intersection gap, matrix size 128 × 128, and the FOV 260 × 210 mm. The TR/TE 3700/80 ms, flip angle 90°, averages 6, with three b values of 0, 100, and 1000 s/mm^2^ . ADC maps were then automatically generated on the basis of a voxelwise calculation. DCE was performed with a 3D spoiled gradient-echo sequence with TR/TE = 5/1.69 ms, flip-angle = 12°, FOV 260 × 260 mm, slice thickness was 3.0 mm with no interslice gap, temporal resolution = 5.7 s seconds, and 32 contrast-enhanced sets of images were acquired sequentially. The data acquisition of the dynamic contrast-enhanced images began simultaneously with the initiation of IV bolus administration of gadopentetate dimeglumine (Magnevist; Berlex, Wayne, NJ) at a flow rate of 4 ml/s, followed by a flush of 20 ml of saline solution.

### Prostate volume estimation

The method for estimation of the total prostate volumes from T2-weighted MR images was reported previously [11] and the ITK-SNAP software (Penn Image Computing and Science Laboratory) was adapted for this manual correction task. Briefly, the entire prostate was semiautomatically segmented on T2-weighted MR images [12] and a radiologist (5 years experience in prostate MRI) reviewed and manually corrected the segmentation results, especially at the base and the apex of the prostate, to ensure accuracy. Finally, the adjusted-PSAD was calculated by dividing PSA concentration by the MR-based prostate volume.

### MR image analysis

Two urogenital radiologists (3 and 5 years of experience, respectively, in prostate imaging) reviewed the images in consensus at a standard Picture Archive and Communication System (PACS) workstation ((Syngo, Siemens Healthcare, Erlangen, Germany). These two readers whom were blinded to initial mp-MR imaging reports and resultant clinical-pathologic outcomes, scored the examinations. The PI-RADS v2 scores were assessed on each of the sequences of T2WI, DWI, and DCE-MRI in turn to provide the overall PI-RADS v2 score [13]. If there were multiple lesions, the PI-RADS v2 score of the index lesion demonstrating the largest size or the most aggressive feature (i.e., extracapsular extension) was assigned to the patient.

### Biopsy procedure and Histopathology

At time of biopsy, first, standardized 12-core transrectal US-guided systematic biopsy was performed by a urologist (who had 4 years of experience with prostate biopsy). Next, targeted biopsy was performed by same operator; these biopsies consisted of at least one additional core per target, the TB were using cognitive registration (cognitive TB [TB-COG]) on the basis of zonal anatomy or imaging landmarks (eg, cysts, remarkable nodules), which was described in a previously published studies [14, 15]. All biopsy cores were immediately fixed in formalin, stained with haematoxylin and eosin (H&E) and underwent routine histopathological evaluation. A Gleason score of ≥ 7 were defined as ‘high-grade prostate cancer’.

### Statistical analysis

As a primary analysis, we considered the statistical associations between the mp-MRI and clinical data with the binary outcome of HGPCa (present/absent). The data were presented as median (interquartile range) or mean (standard deviation), as appropriate. For comparison of continuous variables, the Welch *t* test was used or the Mann-Whitney-Wilcoxon test as a nonparametric alternative. A chi-square or Fisher exact test was applied to compare proportions.

Univariate and multivariate analyses were performed using logistic regression analysis to determine significant predictors of HGPCa. Odd ratios and 95% CIs were determined. The Hosmer-Lemeshow goodness-of-fit test was used to test the quality of the fitted model to the observed data, with a result of *p* > 0.05 considered a good fit. The area under the receiver operating characteristic curve was used to evaluate each predictor and how the model can allow discrimination between patients with and without HGPCa. Area under the curve (AUC) was compared against each other using the DeLong method to determine if a significant difference was present. The statistical analysis was performed using STATA version 9.0 (StataCorp LP, College Station, TX) and Medcalc 15.8 (Medcalc Software bvba, Ostend, Belgium). The nomogram was generated using the R software package (http://www.r-project.org/). An association between the nomogram-based risk-score as well as other parameters with Gleason score (GS) of HGPCa was tested by the Spearman rank correlation analysis. To further evaluate the model’s performance, the nomogram-generated probability was calculated for every patient in the validation cohort then compared with pathology outcomes. A *p* < 0.05 was considered to indicate statistical significance.

## Results

### Patients demographics

For the training cohort, 67 patients (44%) were negative for PCa (benign lesions). Biopsy revealed high-grade PCa in 32 patients (21%) and low-grade PCa in 52 patients (35%). Gleason Score distribution of training cohort was as follows: 3 + 3 = 6 (52 patients), 3 + 4 = 7 (6 patients), 4 + 3 = 7 (8 patients), 4 + 4 = 8 (6 patients), 4 + 5 = 9 (4 patients), 5+ 4 = 10 (4 patients) and 5+ 5 = 10 (4 patients). For the validation patient cohort, 36 of the 74 (48%) were classified as benign lesions. Biopsy revealed high-grade PCa in 18 patients (24%) and low-grade PCa in 20 patients (28%). Gleason Score distribution of validation cohort was as follows: 3 + 3 = 6 (20 patients), 3 + 4 = 7 (4 patients), 4 + 3 = 7 (3 patients), 4 + 4 = 8 (3 patients), 4 + 5 = 9 (4 patients), 5 + 4 = 9 (2 patients) and 5 + 5 = 9 (2 patients). Patient characteristics are detailed in Table 1. The baseline characteristics showed no statistically significant differences between both cohorts.

**Table 1:** Descriptive characteristics of the study population

Variable	Training cohort	Validation cohort	*p* value
Patients, n	151	74	NA
Age, yr, (median; IQR)	63.5; 65–74	64.9;62–73	0.26
tPSA, ng/ml, (median; IQR)	5.7; 4.8–6.7	5.3; 4.2–6.6	0.30
fPSA, ng/ml, (median; IQR)	1.12; 0.41–3.39	1.16; 0.32–4.17	0.21
PSA f/t, (median; IQR)	0.13; 0.06–0.44	0.17; 0.09–0.52	0.16
MRI-based PV, cm^3^ (median; IQR)	46.2; 36.4–59.4	48.2; 33.7–58.1	0.32
Adjusted PSAD, ng/ml/cm3, mean (median; IQR)	0.17; 0.12–0.53	0.16; 0.06–0.47	0.71
DRE nodules yes/no, n (%)	86 (57) / 65 (43)	44 (59) / 30 (41)	0.46
TRUS, Hypoechoic (positive)/Isoechoic (negative)	81 (53) / 70 (47)	42 (56) / 32 (44)	0.56
PI-RADS v2 scores, mean (± SD)	3.3 (±0.9)	3.1 (±1.0)	0.50
Pathological outcomes, n (%)			
High-grade cancer	32 (21)	18 (24)	0.80
Low-grade cancer	52 (35)	20 (28)	
Benign	67 (44)	36 (48)	

*IQR* Interquartile range, *SD* Standard deviation, *NA* Not available, *PSA* Prostate-specific antigen, *MRI* Magnetic resonance imaging, *PV* Prostate volume, *PSAD* Prostate-specific antigen density, *DRE* Digital rectal examination, *TRUS* Transrectal ultrasound, *PI-RADS v2* Prostate Imaging Reporting and Data System version 2

**Table 2:** Univariate and multivariate logistic regression analyses to detect clinically significant prostate cancer

Predictor	Univariate analysis		Multivariate analysis	
	OR (95% CI)	*p* value	OR (95% CI)	*p* value
Age	1.040 (0.893–2.089)	0.021	1.074 (1.008–1.243)	0.031
tPSA	0.040 (0.012–0.089)	0.238	NA	
fPSA	1.342 (0.712–1.993)	0.413	NA	
PSA f/t	1.772 (0.832–2.116)	0.043	NA NA NA	
MRI-based PV	1.112 (1.069–1.157)	0.011	NA	
Adjusted PSAD	6.433 (4.293–8.140)	<0.001	4.711 (3.704–6.313)	0.013
DRE results	0.547 (0.199–1.639)	0.078	NA	
TRUS results	0.961 (0.370–1.826)	0.069	NA	
PI-RADS v2 scores	3.231 (2.173–6.804)	<0.001	2.171 (1.345–3.504)	<0.001

*OR* odds ratio, *CI* confidence interval, *NA* Not available, *PSA* Prostate-specific antigen, *MRI* Magnetic resonance imaging, *PV* Prostate volume, *PSAD* Prostate-specific antigen density, *DRE* Digital rectal examination, *TRUS* Transrectal ultrasound, *PI-RADS v2* Prostate Imaging Reporting and Data System version 2

### Construction of LR model

**Fig. 1:**
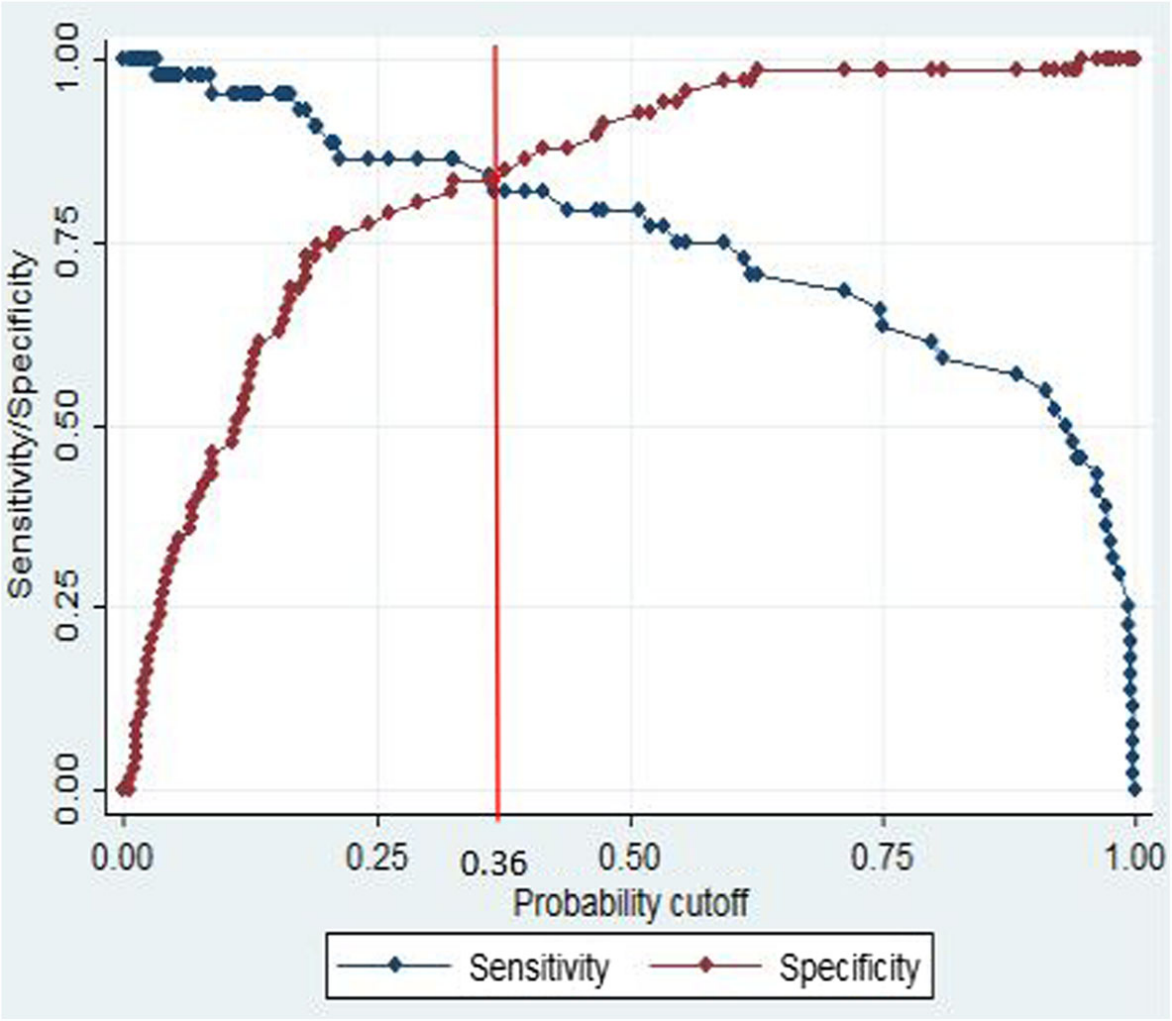
Plot of sensitivity and specificity for logistic regression model. Plot of sensitivity (*red line*) and specificity (*blue line*) as a function of the probability cut points obtained from the logistic model for diagnosising of high-grade prostate cancer. The optimal probability cutoff point was determined to be 0.36

**Fig. 2:**
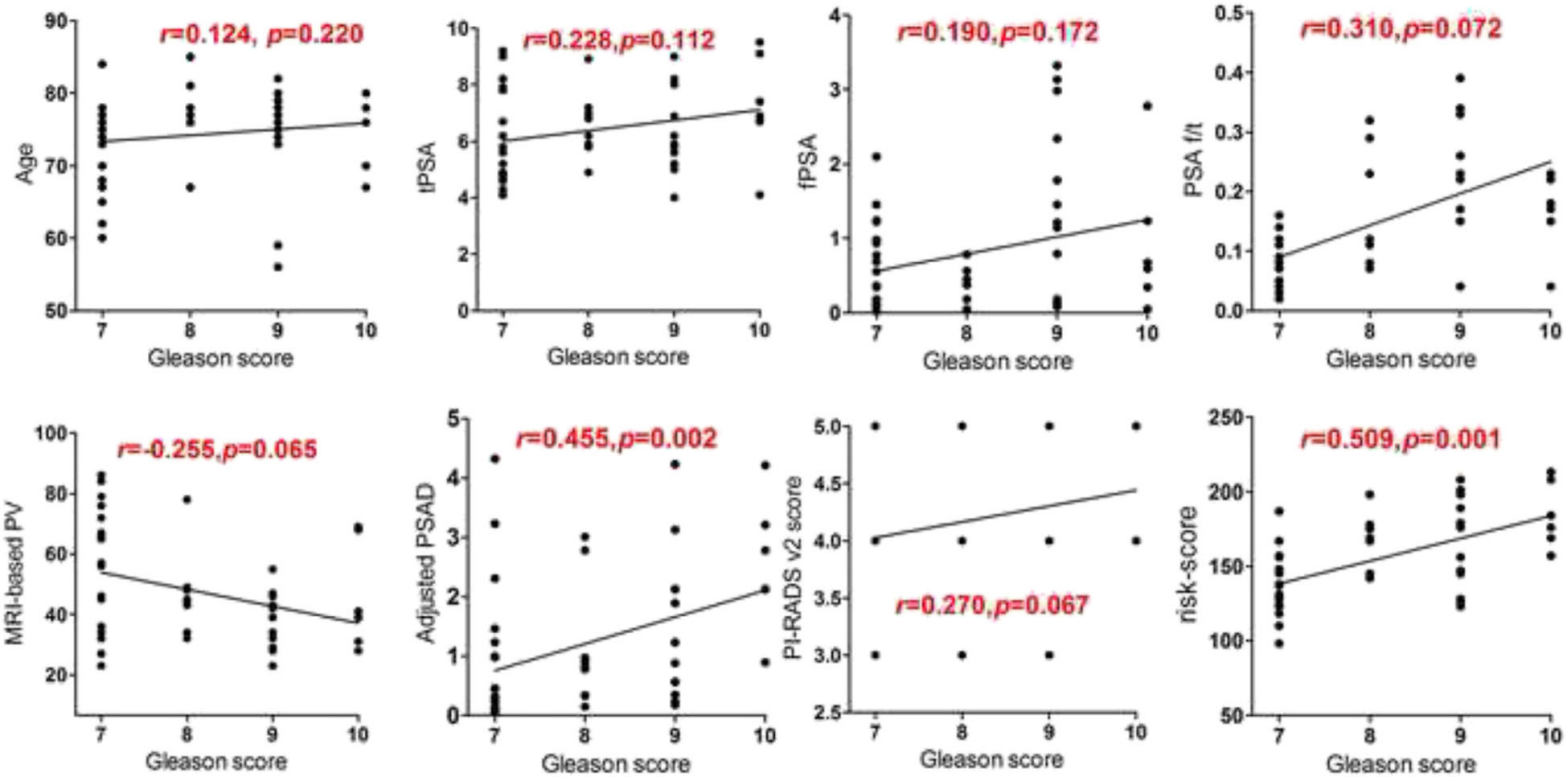
Relationship between all parameters and Gleason scores. Statistically positive correlation were observed between adjusted-PSAD, nomogram-based risk-score with the GS of HGPCa (*r* = 0.455, *P* = 0.002 and *r* = 0.509, *P* = 0.001, respectively), while other parameters found no correlation with GS of HGPCa in PSA gray zone

The univariate logistic regression analysis showed that patient age, PSA f/t ratio, MRI-based PV, adjusted-PSAD, and PI-RADS v2 score were significant predictors of HGPCa in the training cohort. The multivariate logistic regression analysis revealed that the age, PI-RADS v2 score and adjusted-PSAD were independent predictors of HGPCa (Table 2). The cut-off value of the logit was determined based on the ROC curve in consideration of an appropriate tradeoff between the sensitivity and specificity. At the cut-off value of 0.36, i.e., the estimated present of HGPCa before biopsy in this cohort, sensitivity and specificity were 87.3% and 78.4%, respectively (Fig. 1). In addition, the results of the Hosmer-Lemeshow test, which showed a *x* ^2^ value of 2.19 (*p* = 0.31), indicated that the model is almost good fit. For all patients with HGPCa (*n* = 50), adjusted-PSAD and nomogram-based risk-score were positively correlated with the GS of HGPCa (*r* = 0.455, *P =* 0.002 and *r* = 0.509, *P =* 0.001, respectively), while other parameters found no correlation with GS of HGPCa (Fig. 2) in PSA gray zone.

### Validation of LR model

**Fig. 3:**
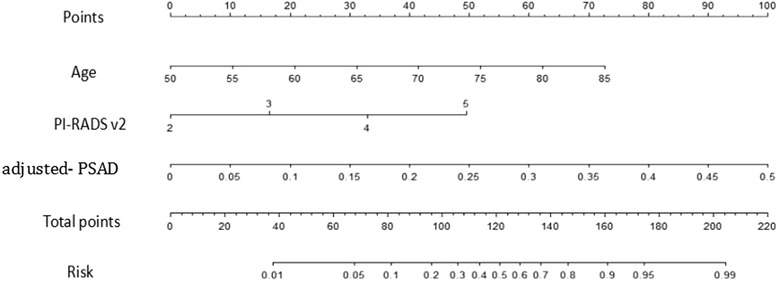
Nomogram shows logistic model for prediction of high-grade prostate cancer. Predictive nomogram for high-grade cancer incorporating age, PI-RADS v2 score, adjusted PSAD. Draw a line upward to number of points in each category. Sum the points and draw a line downward to find the risk of a positive biopsy

**Fig. 4:**
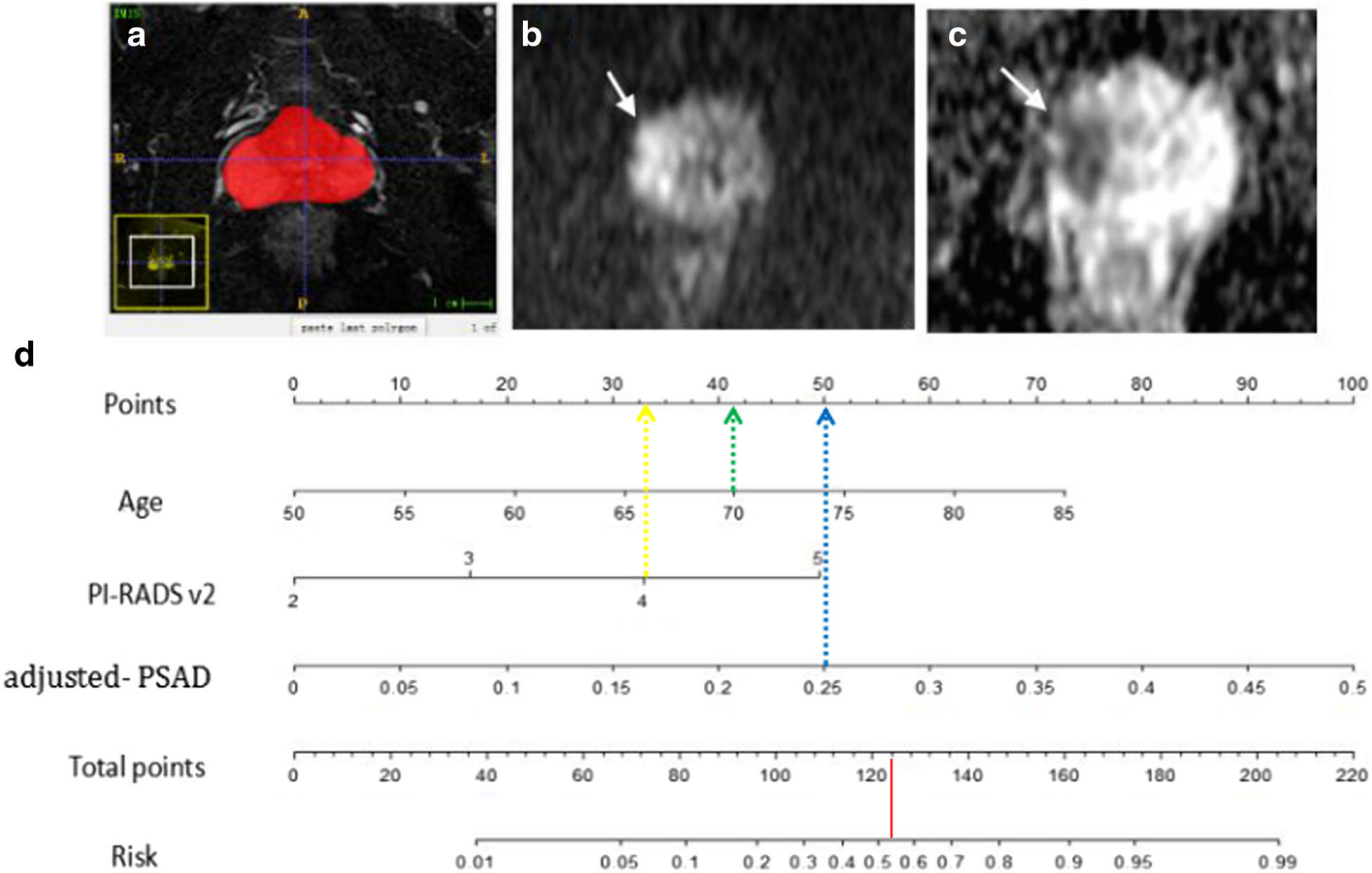
A patient with PSA of 8.6 ng/ml, TRUS-guided biopsy revealed a Gleason 4 + 5 = 9 tumour; (**a**) Labeled segmentation result of entire prostate is seen on T2-weighted axial image by using ITK-SNAP software (Penn Image Computing and Science Laboratory). Based on the segmentation results, the total gland is measured 34 cm^3^ in volume. **b** DWI with *b* = 1000 and (**c**) ADC map show a focal area of diffusion restriction, measuring 1.1 cm in the longest diameter, in the right peripheral zone (*white arrow*). The PI-RADS version 2 score of the DWI-ADC was 4 according to both readers, which is suggestive of a high probability of high-grade cancer cancer. **d** Nomogram for this patient. The corresponding points of the parameters (age, 70 years = 41 points [green line]; PI-RADS v2 score, 4 = 32 points [yellow line]; adjusted PASD 0.25 = 50 points [*blue line*]) yields a total of 123 points. According to nomogram, his probability of having high-grade cancer is 0.53 [*red line*]. Because probability of greater than 0.36 was defined as being compatible with high-grade cancer, nomogram allowed correct prediction of high-grade cancer

The results of ROC-AUC analysis for training set, compare with other parameters are shown in Table 3. The highest AUC for a single risk factor is PI-RADS v2 score (AUC = 0.76). It is notable that in ROC curves, our new model had a larger AUC as compared with other parameters alone. A nomogram was developed using these three independent risk factors (patient age, PI-RADS v2 score and adjusted-PSAD) to forecast HGPCa (Fig. 3). Sample case of the diagnostic use of the nomogram is given in Fig. 4. In validation set, the AUC of the classifier was 0.82 (95% CI, 0.76–0.89), the sensitivity 85.1% and the specificity 76.3%.

**Table 3:** Diagnostic performance of the LR model with other parameters for predicting high grade prostate cancer

Predictor	Area under the Curve (95% CI)	Threshold	Sensitivity (%)	Specificity (%)	PPV	NPV	*p* value
LR model	0.85 (0.79–0.90)	>0.36	87.3	78.4	76.3	90.4	(−)
Age (year)	0.63 (0.50–0.67)	>71.2	72.7	59.4	58.4	73.4	<0.001
tPSA (ng/ml)	0.54 (0.48–0.67)	>7.4	61.2	52.9	51.3	63.5	<0.001
fPSA (ng/ml)	0.52 (0.51–0.69)	>2.1	61.7	60.4	59.4	63.4	<0.001
PSA f/t	0.66 (0.61–0.74)	>0.18	61.1	69.9	59.2	72.9	<0.001
MRI-based PV (cm^3^)	0.64 (0.54–0.72)	<39.4	70.1	60.9	58.8	72.2	<0.001
Adjusted PSAD (ng/ml/cm^3^)	0.74 (0.66–0.79)	>0.16	77.2	60.3	59.3	78.6	0.013
DRE results	0.61 (0.57–0.72)	NA	65.3	59.4	61.5	67.3	<0.001
TRUS results	0.54 (0.51–0.64)	NA	64.1	53.9	51.2	67.9	<0.001
PI-RADS v2 scores	0.76 (0.71–0.84)	>3	78.5	74.2	72.8	79.2	0.018

*LR* Logistic regression, *PSA* prostate-specific antigen, *MRI* Magnetic resonance imaging, *PV* Prostate volume, *PSAD* Prostate-specific antigen density, *DRE* Digital rectal examination, *TRUS* transrectal ultrasound, *PI-RADS v2* Prostate Imaging Reporting and Data System version 2, *PPV* Positive predictive value, *NPV* Negative predictive value

## Discussion

In the PSA gray zone there is still the problem of how to separate the patients who have HGPCa from those who don’t have it. The positive biopsy rate in the diagnostic gray zone of PSA 4–10 ng/ml has been shown to vary across different ethnic groups and countries [16]. In our study, we also proved that the performance of PSA in predicting HGPCa with PSA 4–10 ng/ml was poor (AUC = 0.54). Notably, in these kinds of patient groups up to 80% of biopsies were unnecessary, and therefore, a better risk prediction method specific to these patients is needed.

MRI became the method of choice for detection and staging of PCa [17]. In response, the European Society of Urogenital Radiology (ESUR) drafted guidelines, which have been updated to the PI-RADS v2 recently, by a steering committee including the American College of Radiology (ACR), ESUR and the AdMeTech Foundation [18]. This version assesses the likelihood (probability) of HGPCa and maybe useful for suggesting appropriate patients to active surveillance on a 5-point scale [19]. A meta-analysis that assessed the performance of mp-MRI for detecting prostate cancer demonstrated specificity of 0.88 (95% CI, 0.82–0.92), sensitivity of 0.74 (95% CI, 0.66–0.81) and NPV of 0.64–0.94 [20]. Park et al. [10] reported that the use of PI-RADS v2 might help preoperatively diagnose HGPCa (Sensitivity and specificity were 77.0 and 73.8%, respectively), while, Washino et al. [21] reported that although the PI-RADS score predicts biopsy outcome well, it is difficult to decide which patients can avoid unnecessary prostate biopsies using only the PI-RADS score because of the relatively low PPV.

Through the result of these studies, a model was developed combining PI-RADS v2 score, PSA level, MRI-based PV, adjusted-PSAD, and PSA-related evolutional markers with other independent risk factors, such as age, DRE and TRUS results, into one logistic regression model. The present study shows the AUC of ROC curve for each univariate variable in predicting a biopsy results. PI-RADS v2 score were relatively more important for forecasting HGPCa and were a significant predictor for HGPCa. Compared with PI-RADS v2 score and adjusted-PSAD alone, our newly developed model enlarged AUC from 0.76, 0.74 to 0.85 separately, showing the accuracy for predicting HGPCa was substantially improved. Notably, Given high NPV (90.4%) in this present study, that is to say if the patient’s LR model risk rate blow 0.36, it could be used to reliably rule out HGPCa, obviating the biopsy procedure.

PSA-related evolutional markers including tPSA, PSA f/t ratio are not sufficiently reliable to allow clinical decision making in individual patients [22], which comparable with our results (AUC for PSA f/t ratio was 0.66). The justification for PSAD evaluation was elaborated in some previous study, where it was stated that such marker is better predictor for PCa then PSA level particularly with 4–10 ng/ml [23, 24]. In contrast, our adjusted-PSAD has higher AUC than previous studies. Traditionally, PSA “density,” whereby the PSA value is divided by the prostate volume, estimated from either DRE or TRUS. MRI provides soft-tissue contrast resolution superior to that of transrectal ultrasound so that it can be used for more accurate estimation of prostate volume [25, 26]. Therefore, it is not surprising that the adjusted-PSAD increased the predictive ability of HGPCa and also became a significant predictor for HGPCa.

In current study, although our developed new LR model has achieved high diagnostic performance in detection of HGPCa, the source of false positive and false negative errors should be addressed. Lesion located in PZ, especially central zone (CZ) may not be optimally evaluated using current PZ and TZ criteria. Also, because the CZ commonly exhibits restricted diffusion that is similar in extent to that of tumors, that may potentially yield false-positive or false-negative results. The PZ in men with diffuse prostatitis or marked BPH often exhibits diffusely altered signal characteristics on various sequences, which may pose a diagnostic challenge and yield more false-positive or false-negative results. Furthermore, one particular aspect of PI-RADS v2 for which we have noted particular variability in reader interpretations is scoring of DCE-MRI in PZ of prostate lesions. For example, what exactly constitutes early enhancement and enhancement that is focal and that matches an abnormality on other sequences is unclear. Therefore, once PI-RADS v2 can be applied in a consistent fashion across practices, the system will provide a powerful mechanism for accumulating multicenter data to optimally address these false positive and false negative errors that may change current paradigms for prostate cancer management.

A higher AUC of 0.90 (95% CI, 0.83–0.96) was reported by a study combining traditional clinical risk factors and mRNA levels (HOXC6 and DLX1) to derive a logistic regression model based on a large sample (*n* = 905) [8]. However, to date, only a few biomarkers have reached clinical practice. The main challenge is to validate the performance of the biomarkers in a clinical cohort independently and to demonstrate the clinical utility clearly [27]. Fang et al. [28] developed a ‘PAMD’ score which based on mp-MRI to categorize patients into three risk groups, and the model showed good predictive accuracy for HGPCa (AUC = 0.824). In their study, the prostate volume was determined by TRUS, and the results was not proved by validation cohort.

Histopathologically, the Gleason grading correlates with patient outcome, with higher Gleason scores (GS) indicating more aggressive PCa [29]. Albertsen et al. [30] showed that men with Gleason score (GS) 8–10 PCa have a relatively high probability of dying from PCa within 10 year (12.1%), whereas this risk is minimal for men with low-grade disease. Therefore, we need to predict tumor aggressiveness non-invasively. Litjens et al. [31] found that use of a normalized ADC significantly improved diagnostic accuracy and prediction of cancer aggressiveness, but their assessment was limited to PZ tumors. The results of this study have demonstrated that patients with HGPCa (*n* = 50), the adjusted-PSAD and nomogram-based risk-score were positively correlated with the GS of HGPCa (*r* = 0.455, *P =* 0.002 and *r* = 0.509, *P =* 0.001, respectively). An accurate noninvasive means of both detecting and potentially grading tumors is appealing as a way to enable more-accurate risk stratification of patients, particularly if different treatment options, such as radical prostatectomy or focal therapy, are being considered. In this regard, our results could provide new tool for predicting the aggressiveness of HGPCa before biopsy procedure, especially, nomogram-based risk-score shows relatively strong correlation with GS of HGPCa in PSA gray zone.

Recently, computer-based medical decision support systems have been applied to clinical use for medical diagnosis, decisions, and patient care. Several models—nomograms, risk groupings, artificial neural networks, support vector machines —have been developed to help predict a positive prostate biopsy in men being evaluated for prostate cancer. Nomograms, artificial neural networks and support vector machines improved the accuracy of prediction compared with the individual factors alone. Nomograms are perfect examples of a predictive application that allows a graphical representation of variable interactions and a depiction of their combined effects. Shariat et al. [32] reported that the nomograms have the highest accuracy and the best discriminating characteristics for predicting outcomes in prostate cancer patients.

Patients whose cancer is not clinically significant may be assigned to active surveillance (the lesion is monitored frequently for signs of progression) instead of treatment. In our clinical practice, there is also great potential benefit in the use of mp-MRI for monitoring AS rather than biopsies. As the process of mp-MRI becomes less invasive, greater acceptance amongst patients may follow. Furthermore, with the reliability of mp-MRI to image the entire prostate, it is feasible that patients will feel further reassured that they did not miss any high-grade cancer.

We acknowledge the following limitations. As with any retrospective study, there is risk for selection bias. On mp-MRI we analysed the index lesion, defined as the largest most likely to be cancerous area, this might have been a source of bias in our results. In addition, as mentioned previously, we haven’t compare our new model with other classifiers (e.g., ANN and SVM) in the present study. Finally, our model has not been performed in an external dataset and requires to be tested and verified in more centers with larger samples.

## Conclusion

This study found that the nomogram based mp-MRI for forecasting HGPCa is effective, which could reduce unnecessary prostate biopsies in patients with PSA 4–10 ng/ml and nomogram-based risk-score could provide a more robust parameter of assessing the aggressiveness of HGPCa in PSA gray zone. Future research might indicate that additional parameters could further optimize the diagnosis of HGPCa without contributing to the high unnecessary biopsy rate.

## Abbreviations

ACR: American College of Radiology
ADC: Apparent diffusion coefficient
AUC: Area under the curve
DCE: Dynamic contrast-enhanced imaging
DRE: Digital rectal examination
DWI: Diffusion weighted imaging
ESUR: European Society of Urogenital Radiology
HGPCa: High-grade prostate cancer
LR: Logistic regression
mp-MRI: Multi-parametric magnetic resonance imaging
MRI: Magnetic resonance imaging
PCa: Prostate cancer
PI-RADS v2: Prostate Imaging Reporting and Data System version 2
PSA: Prostate-specific antigen
PSAD: Prostate-specific antigen density
PV: Prostate volume
ROC: Receiver operating characteristic
T2WI: T2 weighted imaging
TB: Target biopsy
US: Ultra-sonographic
